# Perceiving Ourselves and Others: Neural Signatures of Cognitive, Social, and Affective Processes Related to the Perception of Ourselves, Our Body, and the Social World

**DOI:** 10.3390/brainsci12101388

**Published:** 2022-10-14

**Authors:** Michael Schaefer

**Affiliations:** Fakultät Naturwissenschaften, Medical School Berlin, 14197 Berlin, Germany; michael.schaefer@medicalschool-berlin.de

An essential task of any system is to distinguish between itself and its environment, between what is inside and that which is outside. “Draw a distinction and the universe comes into being”, as George Spencer Brown described it decades ago in his book “Laws of form” [1]. Long before the emergence of elaborate forms of consciousness, the first living systems seem to have developed two senses in particular to maintain this difference between their own system and the environment: touch and taste. These two modalities may be among the earliest senses to have evolved and are central to our survival and reproduction. Many unicellular organisms evolved a sense of touch, while sight and hearing evolved millions of years later in multicellular animals. Moreover, the sense of touch (and perhaps the sense of hearing) is the first sense that humans develop before birth. Although the other senses are certainly important as well, the sense of touch may still be particularly important today in establishing our identity and distinguishing it from the environment.

The question of how the brain manages this distinction has been the subject of research for the last decades. In his famous studies, Wilder Penfield demonstrated that we have a map in the primary somatosensory cortex that represents tactile experiences on our bodies’ surface [2], thereby giving us an impression as to how our brain sees the body. Studies in the last decades showed that the role of the somatosensory cortices might be even more interesting, suggesting that these brain regions may be more complex than previously thought. The tactile senses inform us about contact on our body surfaces by an activation of the primary somatosensory cortices [2], but tactile events seen on other’s bodies also seem to engage our somatosensory brain regions. Touch events that we observe in others are important in telling us what is going on around us and how to interpret a social situation, thus contributing to our empathic feelings and thoughts [3]. Hence, the tactile modality and the somatosensory cortices that represent it may be important not only for distinguishing us from the world (and thereby telling us who we are) but also for connecting us with it.

This also becomes clear when one considers the general dual nature of touch. Among our senses, the tactile sense is unique because it represents a fundamental duality in humans. On the one hand, we can use it to explore the world; on the other hand, we receive information when we feel something on our bodies’ surface. Moreover, the tactile sense is unique since it provides us with events that happen directly on our body, while our eyes and ears inform us about stimuli that are remote from our self. Thus, the sense of touch is linked to the outside world, which we can explore with this sense, but it also provides us with information about what is happening on our body by using an image of our body. It is surprising that the brain can be easily perturbed in the latter task, especially by the non-remote nature of the tactile modality [4]. For example, in intriguing experiments, researchers have shown that we can accept artificial body parts as our own limbs, demonstrated by the famous rubber hand illusion [5]. These ownership illusions show that our brains can be fooled in an amazingly fast and simple way by manipulating the integration of sensory information from vision, proprioception, and touch. Remarkably, this illusion is vivid even when we are aware of this manipulation.

Recent studies on touch and body perception have also drawn our attention to clinical perspectives. Studies demonstrate that body experiences or memories can lead to clinical manifestations such as a memory for pain [6]. Furthermore, body perception and the tactile modality seem to play an important role in various clinical conditions. For example, it has been suggested that the somatosensory cortices may be a treatment target for beneficial plasticity in chronic pain [7] or for some mental disorders related to emotional dysregulation [8]. Furthermore, recent studies showed that our body can be used for self-treatment in surprising ways, even when we are aware of it [4]. This has been demonstrated by bio- and neurofeedback, and more recently by studies showing that placebo responses can occur when subjects know they have received a placebo [9,10].

Although we live in a world that has changed dramatically, the brain is still faced with the task of maintaining a coherent self and distinguishing it from other entities. Remarkably, the sense of touch (and perhaps also taste) is still very important in accomplishing this task and attaching oneself to the social world. Thus, touch is still used today to connect with others (“to get in touch”), by shaking hands or by allowing for greater intimacy. The importance of touch in today’s world was exemplified during the COVID-19 pandemic, where it became clear how difficult our social lives become when touch is no longer possible. Moreover, our lives have changed in the last decades via new forms of interaction brought about by the invention of social media with its “electronic hugs”. The distance in social media, which makes it impossible to connect with others through real touch, may contribute to some of the problems in these new technical developments.

The present Special Issue provides contributions to this area of research, describing various problems, challenges, approaches, and results that seek to understand how the brain manages one of the most basic tasks that any system has to solve, namely, maintaining the difference between itself and the environment. These studies and future research will contribute to our understanding of the dense network of interactions between the mind, body, and environment, a network of interactions that we still successfully use to cope with life in an ever-changing social world.

## References

[1] Brown G.S. (1969). Laws of Form.

[2] Penfield W., Rasmussen T. (1950). The Cerebral Cortex of Man. A Clinical Study of Localization of Function.

[3] Bolognini N., Rossetti A., Fusaro M., Vallar G., Miniussi C. (2014). Sharing social touch in the primary somatosensory cortex. Curr. Biol..

[4] Ramachandran V.S., Rogers-Ramachandran D. (1369). Synaesthesia in phantom limbs induced with mirrors. Proc. Biol. Sci..

[5] Botvinick M., Cohen J. (1998). Rubber hands ‘feel’ touch that eyes see. Nature.

[6] Flor H., Nikolajsen L., Jensen T.S. (2006). Phantom limb pain: A case of maladaptive CNS plasticity?. Nat. Rev. Neurosci..

[7] Flor H., Denke C., Schaefer M., Grüsser S. (2001). Effect of sensory discrimination training on cortical reorganisation and phantom limb pain. Lancet.

[8] Kropf E., Syan S.K., Minuzzi L., Frey B.N. (2019). From anatomy to function: The role of the somatosensory cortex in emotional regulation. Braz. J. Psychiatry.

[9] Kaptchuk T.J., Miller F.G. (2018). Open label placebo: Can honestly prescribed placebos evoke meaningful therapeutic benefits?. BMJ.

[10] Sitaram R., Ros T., Stoeckel L., Haller S., Scharnowski F., Lewis-Peacock J., Weiskopf N., Blefari M.L., Rana R.S.M., Oblak E. (2017). Closed-loop brain training: The science of neurofeedback. Nat. Rev. Neurosci..

